# Molecular Diagnosis of Muscular Dystrophy Patients in Western Indian Population: A Comprehensive Mutation Analysis Using Amplicon Sequencing

**DOI:** 10.3389/fgene.2021.770350

**Published:** 2021-12-03

**Authors:** Komal M. Patel, Arpan D. Bhatt, Krati Shah, Bhargav N. Waghela, Ramesh J. Pandit, Harsh Sheth, Chaitanya G. Joshi, Madhvi N. Joshi

**Affiliations:** ^1^ Gujarat Biotechnology Research Centre, Department of Science and Technology, Government of Gujarat, Gandhinagar, India; ^2^ ONE-Centre for Rheumatology and Genetics, Vadodara, India; ^3^ Foundation for Research in Genetics and Endocrinology (FRIGE), Ahmedabad, India

**Keywords:** next generation sequencing (NGS), multiplex ligation-dependent probe amplification (MLPA), duchenne muscular dystrophy (DMD), becker muscular dystrophy (BMD), limb-girdle muscular dystrophies, congenital muscular dystrophies (CMDs)

## Abstract

Muscular Dystrophies (MDs) are a group of inherited diseases and heterogeneous in nature. To date, 40 different genes have been reported for the occurrence and/or progression of MDs. This study was conducted to demonstrate the application of next-generation sequencing (NGS) in developing a time-saving and cost-effective diagnostic method to detect single nucleotide variants (SNVs) and copy number variants (CNVs) in a single test. A total of 123 cases clinically suspected of MD were enrolled in this study. Amplicon panel-based diagnosis was carried out for 102 (DMD/BMD) cases and the results were further screened using multiplex ligation-dependent probe amplification (MLPA). Whilst in the case of LGMD (N = 19) and UMD (N = 2), only NGS panel-based analysis was carried out. We identified the large deletions in 74.50% (76/102) of the cases screened with query DMD or BMD. Further, the large deletion in *CAPN3* gene (N = 3) and known SNV mutations (N = 4) were identified in LGMD patients. Together, the total diagnosis rate for this amplicon panel was 70.73% (87/123) which demonstrated the utility of panel-based diagnosis for high throughput, affordable, and time-saving diagnostic strategy. Collectively, present study demonstrates that the panel based NGS sequencing could be superior over to MLPA.

## Highlights

• Muscular Dystrophies (MDs) are genetically heterogeneous diseases.
• Loss of function mutations in the *DMD* gene causes non-functional dystrophin protein that progresses various MDs.
• The customized amplicon panel consisting of genes targeting 29 MDs was used to detect large deletions in the *DMD* gene and novel deletion in the *CAPN3* gene.
• NGS-based study provides eligibility of patients for currently available treatment such as exon skipping.

## Introduction

Muscular dystrophy is a genetically heterogeneous group of neuromuscular diseases that result in degradation of skeletal muscles, progressive muscle weakness, loss of ambulation, cardiac attack, and respiratory failure (Wang et al., 2019). To date, more than 30 different types of MDs are known and can be classified based on the onset of disease, clinical manifestations, mode of inheritance, and severity of the disease (Gaina et al., 2019). Duchenne Muscular Dystrophy (OMIM # 310200) is the most common, rapidly progressive, and severe neuromuscular disease. It inherits in an X-linked recessive manner affecting 1 in 3,500 male children with onset before 5 years’ age (Wu et al., 2017; Mohammed et al., 2018; Zhang Y. et al., 2019). Becker Muscular Dystrophy (BMD) is a less severe form of the disease caused by the mutation in the *DMD* gene and slow progressive with an incidence rate of 1 in 20,000 male children (Aartsma-Rus et al., 2016; Wu et al., 2017; Mohammed et al., 2018; Kong et al., 2019; Wang et al., 2019). The *DMD* (dystrophin) is a large gene encompassing 79 exons and spanning approximately 2.5 Mb of the genomic DNA. Loss of function mutations in the *DMD* gene causes an impaired dystrophin protein which disturbs the membrane complex and myofiber loss. In contrast, patients with BMD have a shorter or less functioning form of the dystrophin protein, which makes the disease less severe and slow progressive (Wicklund, 2013; Wu et al., 2017; Wang et al., 2019). DMD and BMD are caused due to various mutations like large deletions (60%), SNVs and INDELs (30%), and duplications in the *DMD* gene (5–7%) (Mohammed et al., 2018; Wang et al., 2019).

Limb-Girdle Muscular Dystrophies (LGMDs) are another heterogeneous group of MDs consisting of around 30 subtypes, vary with genetic and clinical characteristics (Iyadurai and Kissel, 2016). LGMDs are progressive and characterized by weakness of the shoulder and pelvic girdle muscles. The incidence rate of LGMDs is approximately 1 in 14,500 to 123,000 (Pegoraro and Hoffman, 2012; Murphy and Straub, 2015; Nallamilli et al., 2018) [ *https://rarediseases.org/rare-diseases/limb-girdle-muscular-dystrophies* , last accessed July 29, 2020.] The inheritance pattern of LGMDs is both autosomal dominant (AD-LGMD) and autosomal recessive (AR-LGMD). AR-LGMDs are more frequent than AD-LGMDs. LGMD associated proteins includes dystrophin-glycoprotein complex (DGC) and play a pivotal role in membrane stability. The mutations in MD genes causes disturbance in DGC proteins that destabilizes the membrane and eventually muscle degradation (Murphy and Straub, 2015).

Congenital Muscular Dystrophies (CMDs) are another group of muscular dystrophies that are also heterogeneous and affect newborns with an incidence of 1:10,000 to 1:50,000. Common symptoms of CMDs include hypotonia, scoliosis, motor delay, and muscle weakness from birth or infancy. Moreover, mutation in multiple genes causes CMDs (Valencia et al., 2013).

Routinely, Multiplex Ligation Dependent Probe Amplification (MLPA) or array-CGH (aCGH) diagnostics tests are being used to detect large CNVs (Deletions/Duplications) in MDs (Zhang K. et al., 2019). The results of these diagnostic tests further requires targeted sequencing to detect SNVs in the DMD/BMD cases. In several cases in which large deletions can be a cause of LGMDs, an aCGH is an exclusive option (Zhang K. et al., 2019). Performing aCGH in all referred cases would be time consuming and expensive. In a developing country like India, cost-effective and a single screening approach to detect CNVs, and SNVs, can be a boon. Furthermore, variety of therapies for DMD patients are available and few are under development, which requires an utmost knowledge of breakpoints for deletions and targeted mutations. Hence, timely and precise diagnosis of MDs helps clinicians to enroll eligible patients for therapy. The diagnosis of specific subtype of MDs using Next-generation sequencing (NGS) can be a timely and affordable approach which improves clinical prognosis (Bello and Pegoraro, 2016; Okubo et al., 2016; Zhang K. et al., 2019). Further, the NGS platforms also identified the novel variants as well as confirmation of hard-to-detect variants (Sheikh and Yokota, 2020). Recent studies suggest that the utilization of a high-throughput method using NGS platform is more suitable for clinical diagnosis (Okubo et al., 2016; Aravind et al., 2019). Moreover, the detection of large duplications is a major challenge for single-point diagnostic strategy (Okubo et al., 2016). In the present study, a total of 123 subjects (including both patients and female carriers) with suspected MDs were evaluated using an amplicon-based panel for its diagnostic specificity to detect CNVs and SNVs. Results of CNV analysis for the *DMD* gene were compared with MLPA. Further, we aimed to identify CNVs and SNVs type of mutation with our customized amplicon panel for different types of muscular dystrophies.

## Materials and Methods

### Sample Collection and Genomic DNA Isolation

A total of 123 unrelated patients suspected of MD [DMD/BMD (N = 82), LGMD (N = 19), and UMD (N = 2)] and possible carriers (N = 20) were enrolled in this study. These cases were recruited in the study through screening camps across the state of Gujarat by collaborative efforts of the Indian Muscular Dystrophy Society (IMDS), Rashtriya Bal Swasthya Karyakram, and Gujarat Biotechnology Research Centre (GBRC). Informed and written consent was derived from the patients and their relatives after Genetic counseling. We have included patients clinically suspected with DMD/BMD with the following indications 1) significantly high serum creatine phosphokinase (CPK- >200 U/L (Aujla and Patel, 2020); 2) difficulty in walking, waddling gait, toe walk, Gower’s sign or loss of ambulation; 3) and progressive muscle weakness. Patients with evident proximal muscle weakness mainly the shoulder girdle and pelvic were included in the study with query LGMD. Uncertain Muscular Dystrophies (UMDs) were included in the study for amplicon sequencing. Blood samples were collected in EDTA vacutainer in a standard blood collection setup. Genomic DNA was extracted from blood samples using the QIAamp DNA Blood Mini Kit (QIAGEN, Germany) as per the manufacturer’s instructions. DNA quantitation was done on Qubit 4 Fluorometer (Thermo Fisher Scientific, IN) using dsDNA BR (broad range) assay kit (Thermo Fisher Scientific, IN). For the data analysis, the baseline was generated using amplicon sequencing of 10 healthy male controls.

### Customized Multi-Gene Panel

In the present study, a custom Ion AmpliSeq™ Panel which covers *DMD, SGCA, SGCB, SGCG, SGCD, CAPN3, ISPD, TCAP, TMEM43, TRIM32, FKRP, MYOT, POMT1, FKTN, POMT2, POMGNT1, DAG1, LMNA, ANO5, LAMA2, COL6A1, COL6A2, COL6A3, FHL1, DYSF, LARGE, TRAPPC11*, and *EMD* genes which are reported earlier and significantly associated with different pathologies of muscular dystrophies was designed. The association of these genes with different phenotypic abnormalities of MDs has been indicated in Supplementary Table S1. The panel comprises a total of 1,312 amplicon primer pools targeting the coding and untranslated regions (UTRs) with 10bp flanking regions of the mentioned genes.

### Targeted Sequencing

A total of 123 cases (includes 102 DMD/BMD/Carrier, 2 UMD, and 19 LGMD) were screened by targeted sequencing using a custom amplicon panel. For each sample, 50 ng of DNA was amplified with custom primer pools using Ion Ampliseq™ HiFi Mix (Thermo Fisher Scientific, IN). This was followed by partial digestion, adaptor + barcode ligation, and library amplification. Libraries were purified using AgencourtAMPure XP (Beckman Coulter, United States). Purified libraries were quantified by Qubit dsDNA HS assay kit (Thermo Fisher Scientific, IN) and then pooled in equimolar concentrations. Emulsion PCR of pooled and diluted libraries was carried out using the Ion OneTouch™ 2 System (Thermo Fisher Scientific, IN) followed by enrichment of template-positive Ion Sphere™ Particles on an Ion OneTouch™ ES system (Thermo Fisher Scientific, IN). Sequencing was carried out on the Ion Proton™ and Ion S5™ systems using Ion PI and Ion 530 chips respectively, with an average depth of 80x.

### MLPA

A total of 102 DMD/BMD/Carrier subjects were screened through MLPA for all exon deletions and duplications in the human *DMD* gene. MLPA was performed using SALSA MLPA kit P034/P035 (MRC-Holland, Netherlands) as per the manufacturer’s instructions. Fragment analysis was performed on the 3500xL Genetic Analyzer (Applied Biosystems, United States) and MLPA data were analysed using Coffalyser Software (MRC-Holland, Netherlands).

### Analysis of Single Nucleotide Variants

Analysis of the raw sequences was performed using Ion Torrent Suite software v5.12 on the Ion torrent server with the incorporated standard pipeline. Variant analysis pipeline includes, signal processing, base calling, quality score assignment, adaptor trimming, PCR duplicate removal, and read alignment to the human reference genome (hg19 genome build). Variants were identified with Torrent Variant Caller plugin software and the Coverage Analysis plugin software obtained coverage analysis. The poor quality and intronic mutations were discarded from the datasets. Annotation of the high-quality variants was performed using the Ion Reporter server system. Clinically known and reported variants like pathogenic or likely pathogenic were identified from the ClinVar database (Landrum et al., 2014). To check strand biases and sequencing errors in the variant calling, alignments were visualized and the presence of mutations in the datasets against the reference genome was confirmed using Integrative Genomics Viewer (IGV) (Robinson et al., 2011). Classification of variants was carried out as per the American College of Medical Genetics and Genomics recommendations (ACMG) for standard interpretation and reporting of sequence variations (Richards et al., 2015).

### Identification of Large Homozygous and Heterozygous Deletions

For identification of large deletion/s, we used a CNV detection workflow available on the Ion Reporter Server system. For the CNV detection workflow, the base line was created using 10 healthy normal individual male samples. This baseline control was used as a reference to analyse CNV in patient samples and female carriers.

### Analysis of Reading Frame

In the *DMD* gene, in-frame and out-of-frame mutation patterns were analyzed with a reading frame checker of online available DMD database [Leiden Muscular Dystrophy Pages. https:// www.dmd.nl/, last accessed July 29, 2020.].

## Results

**FIGURE 1:**
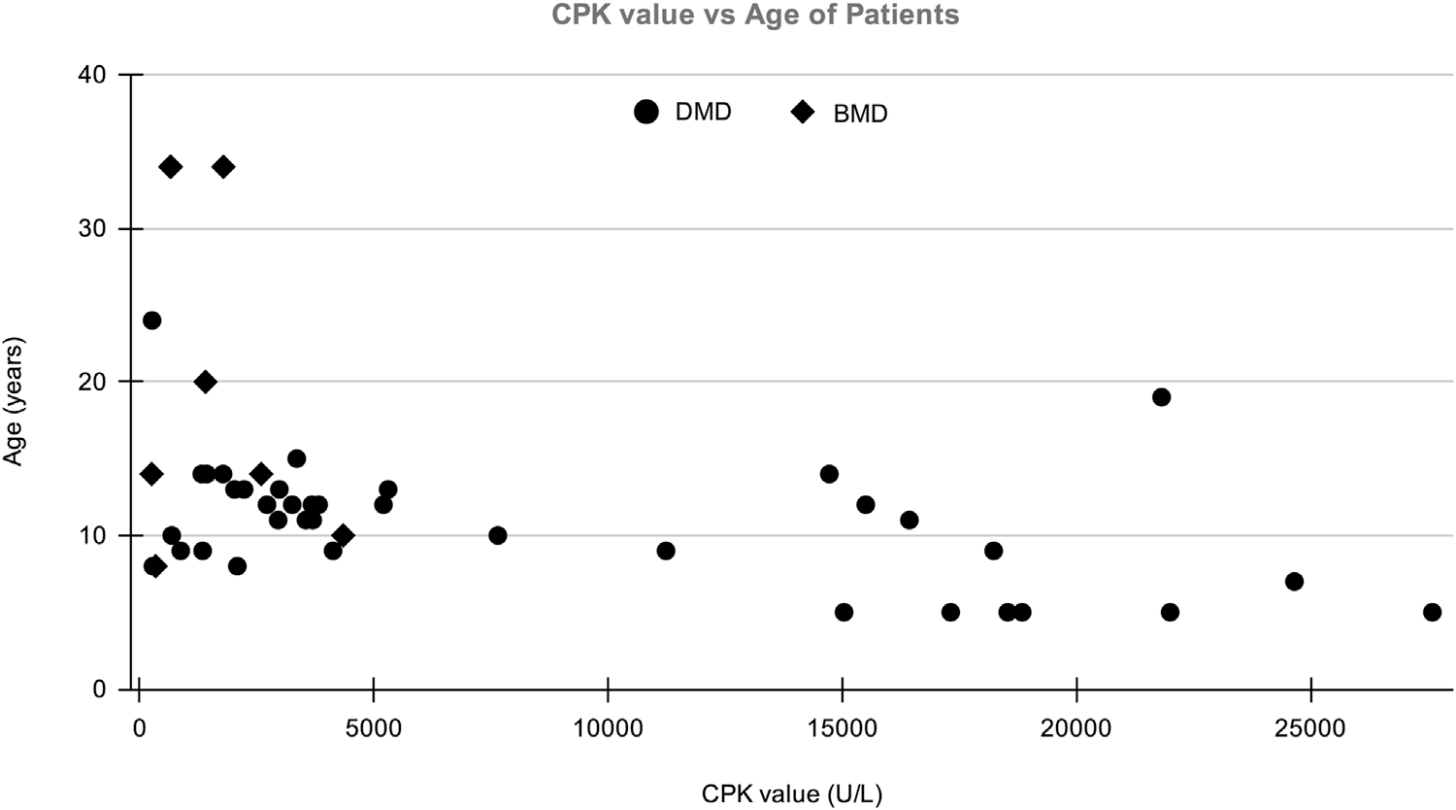
The distribution of patient’s Age vs CPK value in which *X*-axis shows CPK value (U/L) of patients and *Y*-axis shows the age of DMD/BMD patients in our study.

A total of 123 suspected MD patients and suspected female carriers were enrolled in the study. The mean age of onset for DMD and BMD cases was 13 and 22 years, respectively. Average CPK levels in clinically confirmed DMD and BMD cases were 6,551.08 U/L and 1,459.8 U/L, respectively. The normal range of CPK is 20–200 U/L (Aujla and Patel, 2020). The graph of CPK vs age is shown in Figure 1.

### The Large Deletion in DMD/BMD

**FIGURE 2:**
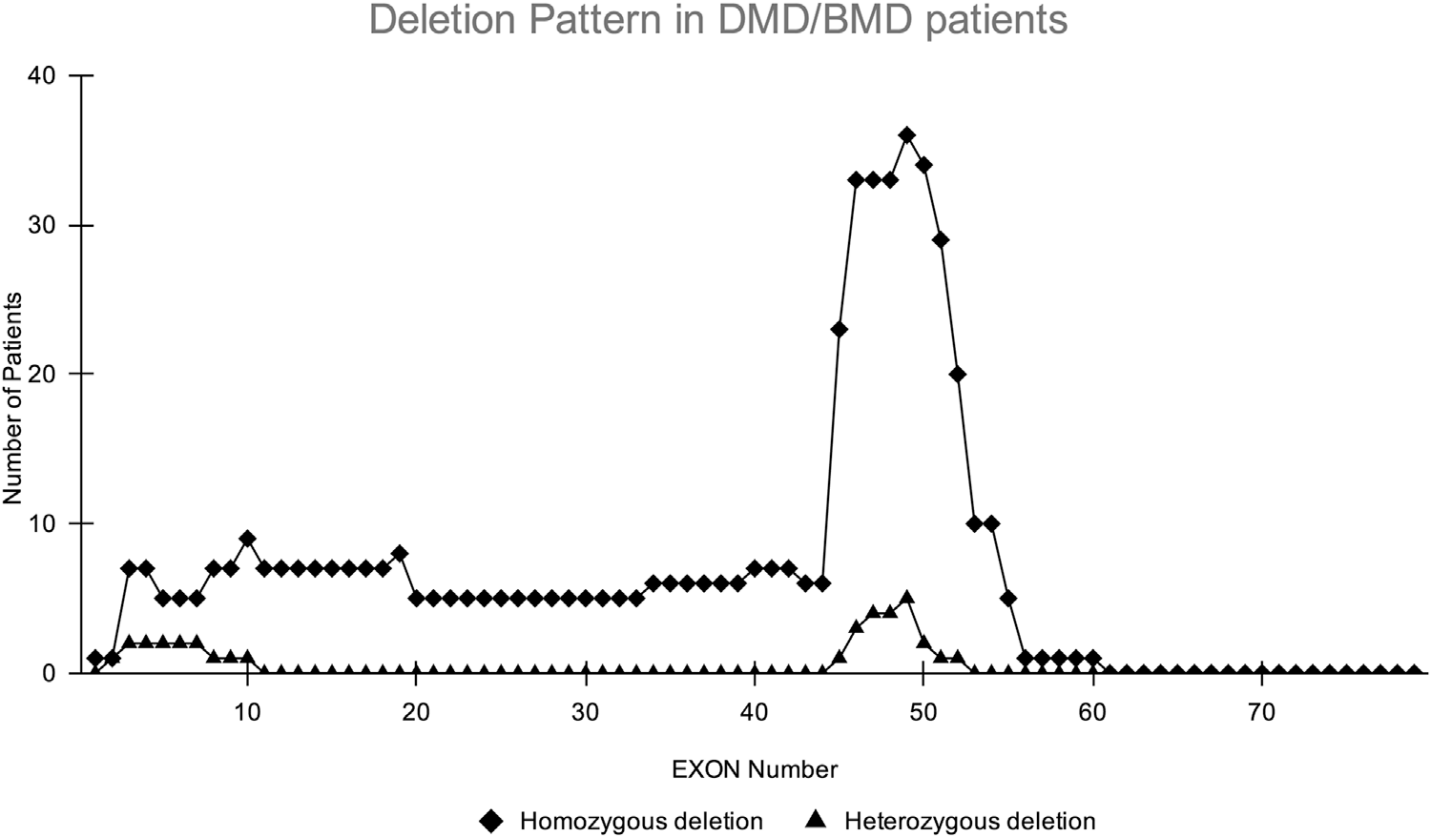
Homozygous and heterozygous deletion (female carrier) pattern in DMD/BMD patients. The *X*-axis shows exon number of *DMD* gene and *Y*-axis shows the number of patients showing deletion in the study. The highest deletions frequency was found in exons 45–52.

**FIGURE 3:**
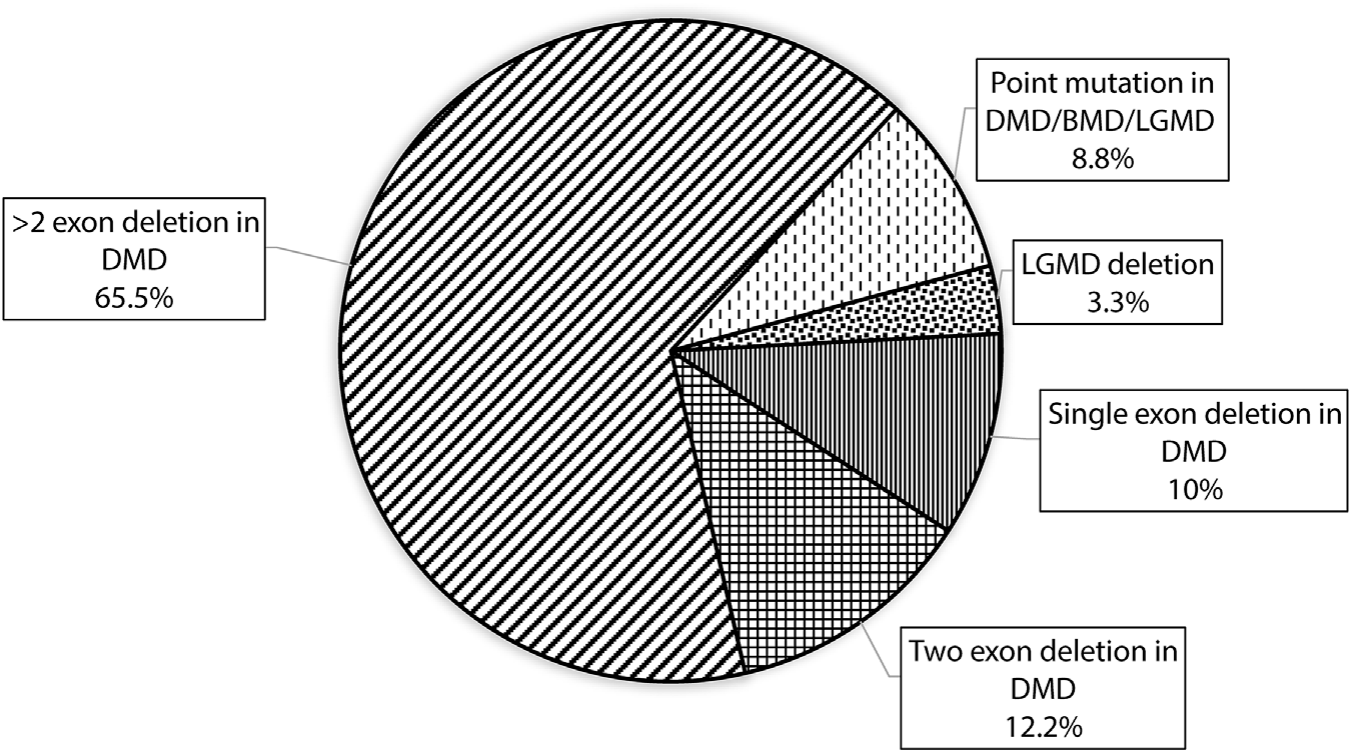
Figure showing the distribution of mutation patterns with designed custom amplicon panel in our study.

CNV analysis of the NGS data of suspected DMD and BMD cases revealed large deletions in 76/102 (74.5%) cases, which included 69 patients and 7 female carriers. As per the reading frame rule, considering only exon deletion, 63/102 (61.76%) and 13/102 (12.75%) cases were categorized into DMD and BMD, respectively. The majority of deletions (78.94%) were in the distal hotspot region (Exon 42–55) and proximal hotspot deletions were between exon 2–19 (10.52%). Two patients showed very large deletion including both proximal and distal hotspot regions. No deletion was observed in exon 61–79. The results of CNV analysis (*DMD* gene) using the NGS panel were concordant with the results obtained using MLPA (Supplementary Table S2). The deletion pattern of all positive cases is depicted in Figure 2. The largest deletion was seen in (exons 1 to 60 (P30) followed by exons 3 to 44 (P41), exons 3 to 42 (P46), and exons 3 to 41 (P55). In the proximal region, the most frequently deleted exon is 10 (10/76, 13.15%) followed by exon 3, and exon 4 (9/76, 11.84%), while in the distal region, the most common deleted exon is 49 (41/76, 53.94%), followed by exon 48 and exon 47 (37/76, 48.68%). Single exon deletion was observed in 9/76 (11.84%) patients, where the most common deletion was observed in exon 45 followed by exon 51. More than one exon deletion was identified in 67/76 patients (88.15%). The distribution of the mutation pattern is shown in Figure 3. In this study, exon 45 to 52 was identified as a major deletion hotspot region. Representative IGV tool image of large deletion is shown in Supplementary Figure S1.

### The Large Deletion in LGMD

Amplicon sequencing analysis of 19 LGMD suspected cases revealed a homozygous deletion of exon 17 to 24 of the *CAPN3* gene in 3 patients (15.78%, 3/19). It was further confirmed by visualizing in IGV (Supplementary Figure S2), which supports the results of CNV workflow. *CAPN3* deletion results are consistent with the clinical presentation of LGMD type 2A disease.

### Single Nucleotide Variation

MLPA and NGS (CNV) negative cases (N = 44) were further considered for SNV analysis where in patient P109, a pathogenic hemizygous mutation was found in the *DMD* gene. This mutation causes a premature translational stop signal at codon 4,729 (p. Arg1577*) of the *DMD* gene, which results in a disrupted protein product. Truncating variants in the DMD gene are known to be pathogenic as per the Clinvar database (Landrum et al., 2014). This variant previously has been reported in individuals affected with DMD (Mah et al., 2011; Yang et al., 2013). In the case of suspected LGMD, mutations in 3 different genes were identified in 4 patients (P99 and P100 are sisters). Two pathogenic mutations were observed in *POMT1* (P83) and *DYSF* (P92) genes and other 3 VUS mutations were observed in *LAMA2* (P60), and *SGCB* (P99 and P100) genes. SNV analysis also revealed a missense Variant of Uncertain Significance (VUS) in 1 UMD and 1 suspected LGMD case in *COL6A2* (P52) and *COL6A1* (P97) gene, respectively (Table 1) which causes Bethlem myopathy 1 (LGMD R22 Collagen6-related) disease as per the Clinvar database (Landrum et al., 2014).

**TABLE 1:** Summary of point mutations in LGMD and UMD cases identified using panel-based NGS sequencing.

Patient ID	CPK total	Age	Locus	Location	Function	Exon	Gene-cDNA	ClinVar’s clinical significance	dbSNP ID	Clinical phenotypesof disease
P52	95	40	chr21:47537326	Exonic	missense	11	*COL6A2*-c.1012C>T	Uncertain significance	rs775751831	LGMD R22 Collagen6-related/Ullrich CMD type 1
P60	NA	9	chr6:129704300	Exonic	missense	35	*LAMA2*-c.4993G>A	Uncertain significance	rs373997222	CMD due to partial LAMA2 deficiency
P83	3,675	18	chr9:134397500	Exonic	missense	19	*POMT1*-c.1958C>T	Pathogenic	rs149682171	LGMD R5 γ-sarcoglycan-related
P92	1,631	28	chr2:71788881	Splice site	unknown	23	*DYSF*-c.2217-1G>T	Pathogenic	rs886044379	LGMD R2 dysferlin-related
P97	653	21	chr21:47419593	Exonic	missense	27	*COL6A1*-c.1763C>T	Uncertain significance	rs759442615	LGMD R22 Collagen6-related/Ullrich CMD type 1
P99	995	32	chr4:52895918	Exonic	missense	3	*SGCB*-c.355A>T	Uncertain significance	rs762412447	LGMD R4 β-sarcoglycan-related
P100	679	34	chr4:52895918	Exonic	missense	3	*SGCB*-c.355A>T	Uncertain significance	rs762412447	LGMD R4 β-sarcoglycan-related
P109	3,673	12	chrX:32398743	Exonic	missense	34	*DMD*-c.4729C>T	Pathogenic	rs863224999	DMD

DMD, duchenne muscular dystrophy; LGMD, Limb-girdle muscular dystrophies; CMD, congenital muscular dystrophy; CPK, creatine phosphokinase; NA, not available.

Note, 2018 Note: LGMDs, were described according to new nomenclature proposed by ENMC, Consortium (Straub et al., 2018) and Bethlem myopathy was described as a type of LGMD (Angelini et al., 2018).

## Discussion

In this study, we showed the utility of an amplicon panel to detect CNVs and SNVs to diagnose a heterogeneous group of MDs in patients and carriers. The accurate diagnosis of different types of muscular dystrophies using a single method such as Sanger sequencing or MLPA is a big challenge due to the complex mutational spectrum. MLPA being a first-line test for the diagnosis of the most common type of MD (DMD/BMD), to detect SNVs sequencing is mandatory. However, Sanger sequencing of the large coding region becomes laborious as well as costly (Wang et al., 2014; Wei et al., 2014). Hence, NGS could be the better alternative in terms of cost, since per base sequencing cost has decreased drastically (Pareek et al., 2011). Also, in cases with LGMDs and CMDs, neuromuscular disease-specific panels at a lower cost can be beneficial in developing countries like India. Previously, many studies have been published for NGS-based approaches for MD (Lim et al., 2011; Wang et al., 2014; Wei et al., 2014; Alame et al., 2016). However, there are very few such studies reported for the Indian population (Aravind et al., 2019; Ganapathy et al., 2019; Polavarapu et al., 2019).

We customized an amplicon panel consisting of genes targeting 29 different types of muscular dystrophies. One of the major objectives of the present study was to detect point mutation and CNVs in suspected, DMD/BMD patients/carriers, LGMD, and CMD using an NGS-based amplicon panel. Our CNV results of the *DMD* gene are consistent with the MLPA results. Our findings support the idea that NGS-based diagnosis methods could be routinly employed as a single diagnostic screening method for the most frequent type of MDs. Further, different MDs can be characterized by genotype and phenotype correlation. Earlier reports highlighted the importance of respective mutations in DMD patients and their mutation-specific therapies (Kohli et al., 2020). Identification of mutation patterns in the Indian cohort could improve the therapeutic management. In the mutation analysis, deletion was observed almost in each exon of *DMD* gene except 61–79 exons, where some deletions are reported in very low frequency in Leiden Open Variation database (LOVD) (Aartsma-Rus et al., 2006). Such as deletions are exon 19–45 (P15), 10–19 (P27 and P108), 1–60 (P30), and in 3–41 (P55). Furthermore, two novel out-of-frame deletions (exon 8–30 in P38 and 46–55 in P61) observed in our study are not reported in the LOVD database. The majority of deletions were observed in the hotspot region of exon 45–52. Interestingly, during sample collection, three patients were enrolled phenotypically as a DMD patients however, our results concluded them as BMD patients with the in-frame mutation. The variants were confirmed in reading frame checker of LOVD database (Takeshima et al., 1994). In SNV analysis, a nonsense variant (c.4729C>T in exon 34) was observed in the *DMD* gene in patient P109. This point mutation has been recorded in the LOVD database as a pathogenic variant, which leads to a premature termination codon (p. Arg1577*) and hence forms a truncated protein. Earlier report suggests that the patients with such mutation are affected with DMD (Esterhuizen et al., 2014). Further, we have found four SNVs, *COL6A2*-c.1012C>T, *LAMA2*-c.4993G>A,*COL6A1*-c.1763C>T, *SGCB*-c.355A>T, reported as VUS in Clinvar database due to their conflicting reports and prediction from various computational tools and require further characterizations. NGS analysis of LGMD patients for CNV analysis revealed a homozygous deletion in the *CAPN3* gene in exon 17 to 24 which is already reported in our previous study (Bhatt et al., 2019). Identification of the same mutation in 3 patients in the current study accelerates the proof of novel variants in our population. Mutation in the *CAPN3* gene leads to the most common form of autosomal recessive LGMD-2A type of Muscular Dystrophy. Deletion in 17–24 exons results in short truncated non-functional CAPN3 protein (Bhatt et al., 2019). *CAPN3* gene regulates the instruction for Calpain-3 enzyme production that enzyme found in sarcomeres structure of muscle cells which are the basic unit for muscle contraction (Kramerova et al., 2007).

Characterization of the mutational landscape in the population may increase the success of current therapeutics and may provide direction to develop novel drug candidates. Antisense oligonucleotide (AON)-mediated exon skipping approach is currently developed to restore reading frame rule which produces partially function protein in DMD patients. FDA approved drugs such as EXONDYS 51 and VYONDYS 53 are commercially available to treat the patient who has a confirmed mutation to skip exon 51 and 53 respectively [http://www.aetna.com/cpb/medical/data/900_999/0911.html, last accessed 29 July 2020.]. The limitation of the present study is the very low frequency of point mutations and therefore further sampling is required to validate such mutation with our panel. Moreover, from a total of 123 cases, no mutation is detected in 36 cases suggesting further testing is required for other neuromuscular diseases which may rule out in our panel.

## Conclusion

In conclusion, our finding showed the NGS platform could be a future diagnostic tool for identifying disease-causing mutation/s in different Muscular dystrophies, which are currently diagnosed using multiple methods. The analysis of CNV in the *DMD* gene concludes that our custom panel is superior to the MLPA method. NGS-based diagnosis is not only time-saving but also cost-effective method when compared with traditional testing strategies.

## Data Availability

The datasets presented in this study can be found in online repositories https://www.ncbi.nlm.nih.gov/sra?linkname=bioproject_sra_all&from_uid=692346. Accession number is PRJNA692346.

## Supplementary Material

Supplementary Figure S1 Integrative Genomics Viewer (IGV) snapshot depicting a representative image of Ion Reporter software (v5.12) CNV analysis results in DMD gene where red bar showing the deleted region of exon 3-4 in the patient.

Supplementary Figure S2 Integrative Genomics Viewer (IGV) snapshot depicting a representative image of Ion Reporter software (v5.12) CNV analysis results in CAPN3 gene where red bar showing the deleted region of exon 17-24 in the patient.

## Glossary

ANO5: anoctamin 5
CAPN3: calpain 3
COL6A1: collagen type VI alpha 1 chain
COL6A2: collagen type VI alpha 2 chain
COL6A3: collagen type VI alpha 3 chain
DAG1: dystroglycan 1
DMD: Duchenne Muscular Dystrophy
DYSF: dystrophy-associated fer-1-like protein
EMD: Emerin
FHL1: Four-and-a-Half Lim Domains 1
FKRP: fukutin related protein
FKTN: fukutin
ISPD: isoprenoid synthase domain-containing protein
LMNA: lamin A/C
LAMA2: laminin subunit alpha 2
LARGE: LARGE xylosyl- and glucuronyltransferase 1
MYOT: myotilin
NGS: Next Generation Sequencing
POMT1: protein O-mannosyltransferase 1
POMT2: protein O-mannosyltransferase 2
POMGNT1: protein O-linked mannose N-acetylglucosaminyltransferase 1 (beta 1,2-)
SGCA: Sarcoglycan Alpha
SGCB: Sarcoglycan Beta (43 kDa Dystrophin-Associated Glycoprotein)
SGCG: Sarcoglycan Gamma
SGCD: Sarcoglycan Delta
TCAP: Titin-Cap associated protein
TMEM43: Transmembrane Protein 43
TRAPPC11: trafficking protein particle complex 11
TRIM32: tripartite motif containing 32
VUS: Variants of Uncertain Significance
