# Supplementary material for: Molecular Diagnosis of Muscular Dystrophy Patients in Western Indian Population: A Comprehensive Mutation Analysis Using Amplicon Sequencing
Source: Front Genet. 2021 Dec 3;12:770350. doi: 10.3389/fgene.2021.770350 (PMC8679082; doi:10.3389/fgene.2021.770350)
Supplement: Supplementary file 1 [file Table1.DOCX]

| **Table:1** Association of Genes with Muscular Dystrophies reported in human Phenotype Ontology (HPO) | | | |
| --- | --- | --- | --- |
| **Genes** | **Gene Name** | **Chromosomal Location** | **Associated Diseases** |
| *ANO5* | ANOCTAMIN 5 | 11p14.3 | (ORPHA:206549) - Anoctamin-5-related Limb-girdle Muscular Dystrophy R12  (OMIM:611307) - Muscular Dystrophy, Limb-girdle, Type 2l  (OMIM:613319) - Miyoshi Muscular Dystrophy 3  (OMIM:166260) - Gnathodiaphyseal Dysplasia  (ORPHA:399096) - Distal Anoctaminopathy  (ORPHA:53697) - Gnathodiaphyseal Dysplasia |
| *CAPN3* | CALPAIN, LARGE POLYPEPTIDE L3 | 15q15.1 | (ORPHA:267) - Calpain-3-related Limb-girdle Muscular Dystrophy R1  (OMIM:253600) - Muscular Dystrophy, Limb-girdle, Type 2a  (OMIM:618129) - Muscular Dystrophy, Limb-girdle, Autosomal Dominant 4 |
| *COL6A1* | COLLAGEN, TYPE VI, ALPHA-1 | 21q22.3 | (ORPHA:75840) - Congenital Muscular Dystrophy, Ullrich Type  (OMIM:254090) - Ullrich Congenital Muscular Dystrophy 1  (OMIM:158810) - Bethlem Myopathy  (ORPHA:610) - Bethlem Myopathy |
| *COL6A2* | COLLAGEN, TYPE VI, ALPHA-2 | 21q22.3 | (ORPHA:75840) - Congenital Muscular Dystrophy, Ullrich Type  (OMIM:254090) - Ullrich Congenital Muscular Dystrophy 1  (OMIM:158810) - Bethlem Myopathy  (ORPHA:610) - Bethlem Myopathy  (OMIM:255600) - Myosclerosis, Autosomal Recessive |
| *COL6A3* | COLLAGEN, TYPE VI, ALPHA-3 | 2q37.3 | (ORPHA:464440) - Primary Dystonia, Dyt27 Type  (OMIM:616411) - Dystonia 27  (ORPHA:75840) - Congenital Muscular Dystrophy, Ullrich Type  (OMIM:254090) - Ullrich Congenital Muscular Dystrophy 1  (OMIM:158810) - Bethlem Myopathy  (ORPHA:610) - Bethlem Myopathy |
| *DAG1* | DYSTROPHIN-ASSOCIATED GLYCOPROTEIN 1 | 3p21.31 | (ORPHA:370997) - Muscle-eye-brain Disease with Bilateral Multicystic Leucodystrophy  (ORPHA:899) - Walker-warburg Syndrome  (ORPHA:280333) - Alpha-dystroglycan-related Limb-girdle Muscular Dystrophy R16  (OMIM:613818) - Muscular Dystrophy-dystroglycanopathy (limb-girdle), Type C, 7  (OMIM:616538) - Muscular Dystrophy-dystroglycanopathy (congenital With Brain And Eyeanomalies |
| *DMD* | DYSTROPHIN | Xp21.2 | (ORPHA:98895) - Becker Muscular Dystrophy  (ORPHA:154) - Familial Isolated Dilated Cardiomyopathy  (OMIM:302045) - Cardiomyopathy, Dilated, 3b  (ORPHA:777) - X-linked Non-syndromic Intellectual Disability  (OMIM:300376) - Muscular Dystrophy, Becker Type  (OMIM:310200) - Muscular Dystrophy, Duchenne Type  (ORPHA:98896) - Duchenne Muscular Dystrophy |
| *DYSF* | DYSFERLIN | 2p13.2 | (OMIM:253601) - Muscular Dystrophy, Limb-girdle, Type 2b  (ORPHA:178400) - Distal Myopathy with Anterior Tibial Onset  (ORPHA:45448) - Miyoshi Myopathy  (OMIM:254130) - Miyoshi Muscular Dystrophy 1  (OMIM:606768) - Myopathy, Distal, With Anterior Tibial Onset  (ORPHA:268) - Dysferlin-related Limb-girdle Muscular Dystrophy R2 |
| *EMD* | EMERIN | Xq28 | (ORPHA:98863) - X-linked Emery-dreifuss Muscular Dystrophy  (OMIM:310300) - Emery-dreifuss Muscular Dystrophy, X-linked |
| *FHL1* | Four And A Half LIM Domains 1 | Xq27.2 | (OMIM:300280) - Uruguay Faciocardiomusculoskeletal Syndrome  (OMIM:300696) - Myopathy, X-linked, With Postural Muscle Atrophy  (OMIM:300717) - Myopathy, Reducing Body, X-linked, Early-onset, Severe  (ORPHA:98863) - X-linked Emery-dreifuss Muscular Dystrophy  (OMIM:300718) - Myopathy, Reducing Body, X-linked, Childhood-onset  (OMIM:300695) - Scapuloperoneal Myopathy, X-linked Dominant |
| *FKRP* | FUKUTIN-RELATED PROTEIN | 19q13.32 | (OMIM:613153) - Muscular Dystrophy-dystroglycanopathy (congenital with Brain and Eyeanomalies), Type A, 5  (OMIM:606612) - Muscular Dystrophy-dystroglycanopathy (congenital with Or Without  (ORPHA:899) - Walker-warburg Syndrome  (ORPHA:588) - Muscle-eye-brain Disease  (ORPHA:370959) - Congenital Muscular Dystrophy with Cerebellar Involvement  (OMIM:607155) - Muscular Dystrophy-dystroglycanopathy (limb-girdle), Type C, 5  (ORPHA:34515) - Fkrp-related Limb-girdle Muscular Dystrophy R9  (ORPHA:370980) - Congenital Muscular Dystrophy Without Intellectual Disability  (OMIM:236670) - Muscular Dystrophy-dystroglycanopathy (congenital With Brain And Eye  (ORPHA:370968) - Congenital Muscular Dystrophy With Intellectual Disability |
| *FKTN* | FUKUTIN | 9q31.2 | (OMIM:613152) - Muscular Dystrophy-dystroglycanopathy (congenital Without Mental Retardation),type B, 4  (OMIM:611615) - Cardiomyopathy, Dilated, 1x  (ORPHA:899) - Walker-warburg Syndrome  (ORPHA:588) - Muscle-eye-brain Disease  (OMIM:253800) - Muscular Dystrophy-dystroglycanopathy (congenital With Brain And Eyeanomalies), Type A, 4  (ORPHA:272) - Congenital Muscular Dystrophy, Fukuyama Type  (OMIM:611588) - Muscular Dystrophy, Limb-girdle, Type 2m  (ORPHA:154) - Familial Isolated Dilated Cardiomyopathy  (ORPHA:370980) - Congenital Muscular Dystrophy Without Intellectual Disability  (OMIM:236670) - Muscular Dystrophy-dystroglycanopathy (congenital With Brain And Eye |
| *LAMA2* | LAMININ, ALPHA-2 | 6q22.33 | (OMIM:607855) - Muscular Dystrophy, Congenital, Merosin Deficient Or Partially Deficient  (ORPHA:258) - Laminin Subunit Alpha 2-related Congenital Muscular Dystrophy  (OMIM:618138) - Muscular Dystrophy, Limb-girdle, Autosomal Recessive 23 |
| *LARGE* | ACETYLGLUCOSAMINYL-TRANSFERASE-LIKE PROTEIN | 22q12.3 | (ORPHA:899) - Walker-warburg Syndrome  (ORPHA:588) - Muscle-eye-brain Disease  (OMIM:608840) - Muscular Dystrophy, Congenital, Type 1d  (OMIM:236670) - Muscular Dystrophy-dystroglycanopathy (congenital With Brain And Eye  (ORPHA:370968) - Congenital Muscular Dystrophy With Intellectual Disability  (OMIM:613154) - Muscular Dystrophy-dystroglycanopathy (congenital With Brain And Eyeanomalies), |
| *LMNA* | LAMIN A/C | 1q22 | (ORPHA:157973) - Congenital Muscular Dystrophy Due To Lmna Mutation  (OMIM:275210) - Tight Skin Contracture Syndrome, Lethal  (ORPHA:79084) - Familial Partial Lipodystrophy, Köbberling Type  (ORPHA:740) - Hutchinson-gilford Progeria Syndrome  (ORPHA:2229) - Dilated Cardiomyopathy-hypergonadotropic Hypogonadism Syndrome  (ORPHA:98855) - Autosomal Recessive Emery-dreifuss Muscular Dystrophy  (OMIM:151660) - Lipodystrophy, Familial Partial, Type 2  (ORPHA:98853) - Autosomal Dominant Emery-dreifuss Muscular Dystrophy  (ORPHA:90153) - Mandibuloacral Dysplasia With Type A Lipodystrophy  (OMIM:181350) - Emery-dreifuss Muscular Dystrophy, Autosomal Dominant  (OMIM:610140) - Heart-hand Syndrome, Slovenian Type  (OMIM:605588) - Charcot-marie-tooth Disease, Axonal, Type 2b1  (OMIM:613205) - Muscular Dystrophy, Congenital, Lmna-related  (OMIM:212112) - Malouf Syndrome  (ORPHA:280365) - Autosomal Semi-dominant Severe Lipodystrophic Laminopathy  (OMIM:248370) - Mandibuloacral Dysplasia  (OMIM:115200) - Cardiomyopathy, Dilated, 1a  (ORPHA:79474) - Atypical Werner Syndrome  (OMIM:176670) - Hutchinson-gilford Progeria Syndrome  (OMIM:616516) - Emery-dreifuss Muscular Dystrophy 3, Autosomal Recessive  (ORPHA:2348) - Familial Partial Lipodystrophy, Dunnigan Type  (ORPHA:363618) - Lmna-related Cardiocutaneous Progeria Syndrome  (ORPHA:300751) - Familial Dilated Cardiomyopathy With Conduction Defect Due To Lmna Mutation  (ORPHA:168796) - Heart-hand Syndrome, Slovenian Type  (ORPHA:1662) - Restrictive Dermopathy |
| *POMGNT1* | PROTEIN O-MANNOSE  BETA-1,2-N-ACETYLGLUCOSAMINYL-TRANSFERASE | 1p34.1 | (ORPHA:899) - Walker-warburg Syndrome  (OMIM:253280) - Muscular Dystrophy-dystroglycanopathy (congenital with Brain and Eye  (ORPHA:588) - Muscle-eye-brain Disease  (ORPHA:370959) - Congenital Muscular Dystrophy with Cerebellar Involvement  (OMIM:613151) - Muscular Dystrophy-dystroglycanopathy (congenital with Mental Retardation), type B, 3  (ORPHA:791) - Retinitis Pigmentosa  (OMIM:613157) - Muscular Dystrophy-dystroglycanopathy (limb-girdle), Type C, 3  (OMIM:617123) - Retinitis Pigmentosa 76 |
| *POMT1* | PROTEIN O-MANNOSYL-TRANSFERASE 1 | 9q34.13 | (OMIM:609308) - Muscular Dystrophy, Limb-girdle, Type 2k  (ORPHA:86812) - Pomt1-related Limb-girdle Muscular Dystrophy R11  (ORPHA:899) - Walker-warburg Syndrome  (ORPHA:588) - Muscle-eye-brain Disease  (ORPHA:370959) - Congenital Muscular Dystrophy with Cerebellar Involvement  (ORPHA:370980) - Congenital Muscular Dystrophy Without Intellectual Disability  (OMIM:236670) - Muscular Dystrophy-dystroglycanopathy (congenital with Brain and Eye  (ORPHA:370968) - Congenital Muscular Dystrophy with Intellectual Disability  (OMIM:613155) - Muscular Dystrophy-dystroglycanopathy (congenital With Mental Retardation),ty |
| *POMT2* | PROTEIN O-MANNOSYL-TRANSFERASE 2 | 14q24.3 | (OMIM:613158) - Muscular Dystrophy-dystroglycanopathy (limb-girdle), Type C, 2  (ORPHA:899) - Walker-warburg Syndrome  (ORPHA:588) - Muscle-eye-brain Disease  (ORPHA:370959) - Congenital Muscular Dystrophy with Cerebellar Involvement  (ORPHA:206559) - Pomt2-related Limb-girdle Muscular Dystrophy R14  (OMIM:613156) - Muscular Dystrophy-dystroglycanopathy (congenital With Mental Retardation), type B, 2  (OMIM:613150) - Muscular Dystrophy-dystroglycanopathy (congenital With Brain And Eyeanomalies), Type A, 2  (OMIM:236670) - Muscular Dystrophy-dystroglycanopathy (congenital With Brain And Eye  (ORPHA:370968) - Congenital Muscular Dystrophy With Intellectual Disability |
| *SGCA* | SARCOGLYCAN, ALPHA | 17q21.33 | (ORPHA:62) - Alpha-sarcoglycan-related Limb-girdle Muscular Dystrophy R3  (OMIM:608099) - Muscular Dystrophy, Limb-girdle, Type 2d |
| *SGCB* | SARCOGLYCAN, BETA | 4q12 | (OMIM:604286) - Muscular Dystrophy, Limb-girdle, Type 2e  (ORPHA:119) - Beta-sarcoglycan-related Limb-girdle Muscular Dystrophy R4 |
| *SGCD* | SARCOGLYCAN, DELTA | 5q33.2-q33.3 | (OMIM:601287) - Muscular Dystrophy, Limb-girdle, Type 2f  (ORPHA:219) - Delta-sarcoglycan-related Limb-girdle Muscular Dystrophy R6  (ORPHA:154) - Familial Isolated Dilated Cardiomyopathy  (OMIM:606685) - Cardiomyopathy, Dilated, 1l |
| *SGCG* | SARCOGLYCAN, GAMMA | 13q12.12 | (OMIM:253700) - Muscular Dystrophy, Limb-girdle, Type 2c  (ORPHA:353) - Gamma-sarcoglycan-related Limb-girdle Muscular Dystrophy R5 |
| *TCAP* | TITIN-CAP | 17q12 | (ORPHA:154) - Familial Isolated Dilated Cardiomyopathy  (OMIM:607487) - Cardiomyopathy, Dilated, 1n  (OMIM:601954) - Muscular Dystrophy, Limb-girdle, Type 2g |
| *TMEM43* | TRANSMEMBRANE PROTEIN 43 | 3p25.1 | (OMIM:604400) - Arrhythmogenic Right Ventricular Dysplasia, Familial, 5  (OMIM:614302) - Emery-dreifuss Muscular Dystrophy 7, Autosomal Dominant  (ORPHA:98853) - Autosomal Dominant Emery-dreifuss Muscular Dystrophy |
| *TRAPPC11* | TRAFFICKING PROTEIN PARTICLE COMPLEX, SUBUNIT 11 | 4q35.1 | (ORPHA:869) - Triple A Syndrome  (ORPHA:369847) - Intellectual Disability-hyperkinetic Movement-truncal Ataxia Syndrome  (OMIM:615356) - Muscular Dystrophy, Limb-girdle, Type 2s  (ORPHA:369840) - Trappc11-related Limb-girdle Muscular Dystrophy R18 |
| *TRIM32* | TRIPARTITE MOTIF-CONTAINING PROTEIN 32 | 9q33.1 | (OMIM:254110) - Muscular Dystrophy, Limb-girdle, Type 2h  (ORPHA:110) - Bardet-biedl Syndrome  (OMIM:615988) - Bardet-biedl Syndrome 11  (ORPHA:1878) - Trim32-related Limb-girdle Muscular Dystrophy R8 |
