# Supplementary figures and images for: Molecular Diagnosis of Muscular Dystrophy Patients in Western Indian Population: A Comprehensive Mutation Analysis Using Amplicon Sequencing
Source: Front Genet. 2021 Dec 3;12:770350. doi: 10.3389/fgene.2021.770350 (PMC8679082; doi:10.3389/fgene.2021.770350)

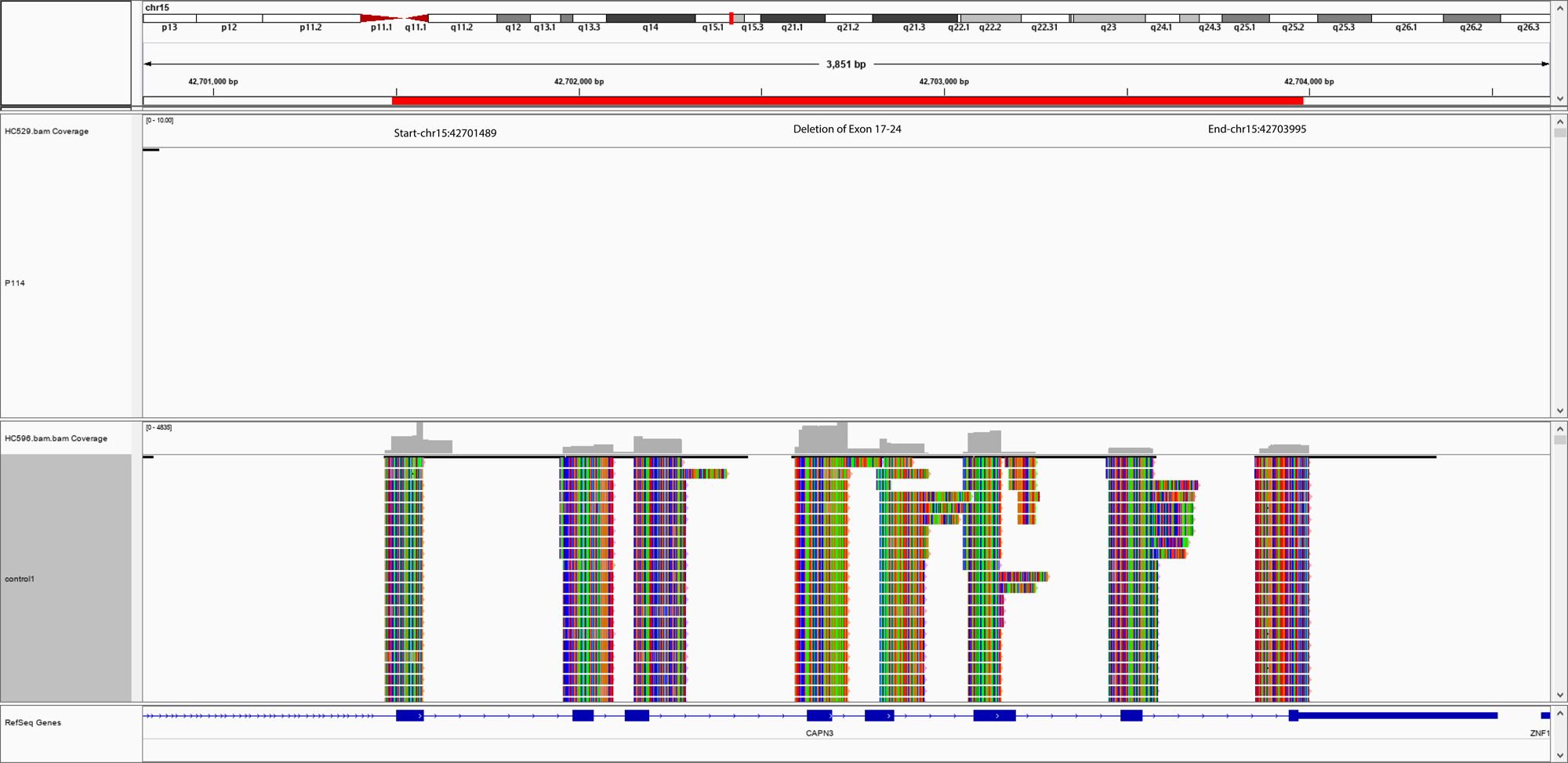

Supplement: Supplementary file 2 [file Image2.TIF]

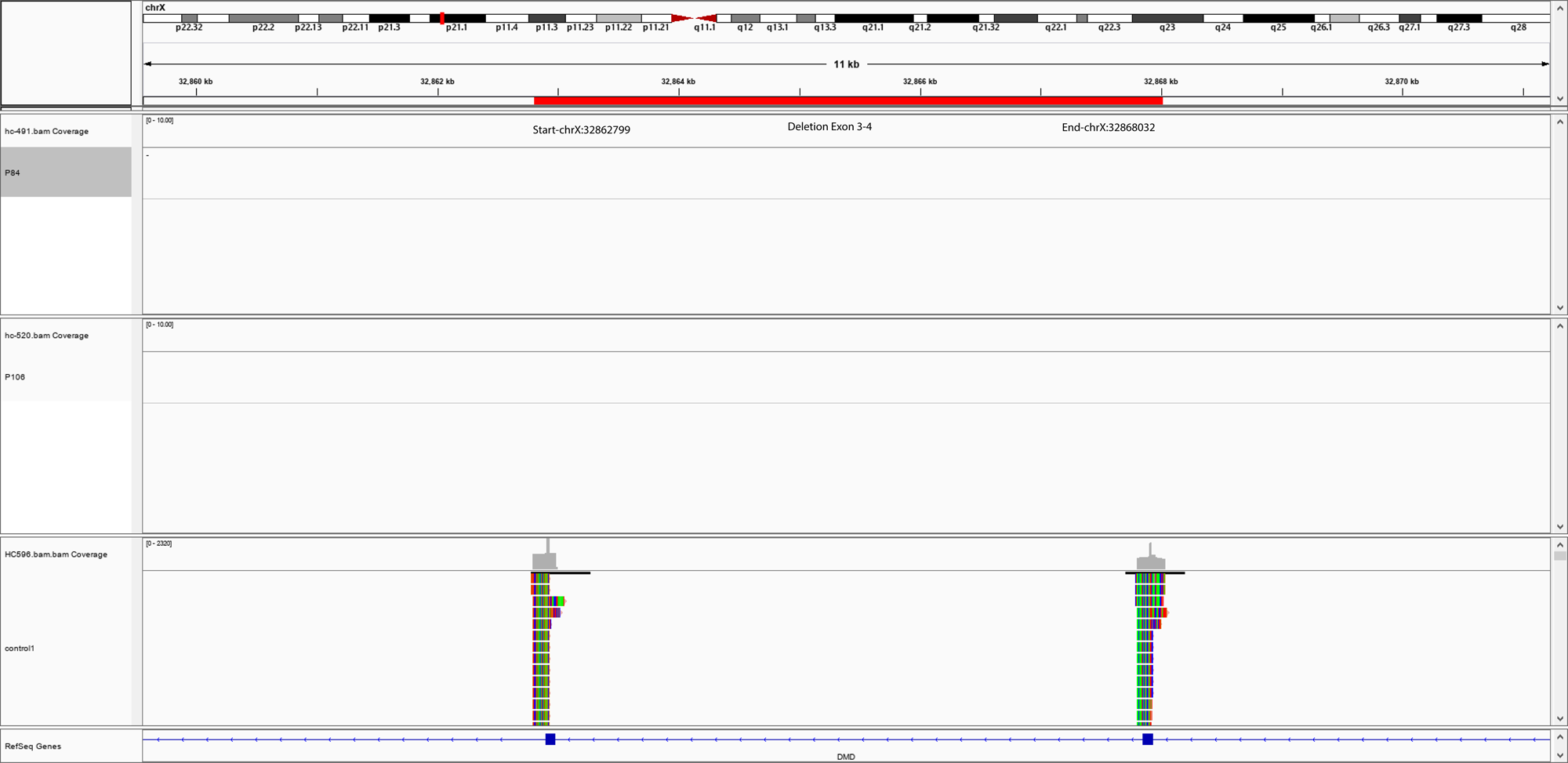

Supplement: Supplementary file 3 [file Image1.TIF]
