# Supplementary material for: Molecular Diagnosis of Muscular Dystrophy Patients in Western Indian Population: A Comprehensive Mutation Analysis Using Amplicon Sequencing
Source: Front Genet. 2021 Dec 3;12:770350. doi: 10.3389/fgene.2021.770350 (PMC8679082; doi:10.3389/fgene.2021.770350)
Supplement: Supplementary file 4 [file Table2.DOCX]

| **Table:2** The clinical details of patients with comparative results of MLPA and NGS test for large deletion | | | | | | | | | |
| --- | --- | --- | --- | --- | --- | --- | --- | --- | --- |
| **Patient ID** | **CPK (U/L)** | **Age**  **(years)** | **Gender** | **Proband/Carrier** | **Genotype of disease** | **NGS CNV results** | **MLPA results** | **Reading frame pattern** | **Breakpoints** |
| P1 | 22000 | 5 | Male | P | DMD | Homo DelEx:44 | Homo DelEx:44 | OUT-OF-FRAME | Xp21.1(32173403-32235295)x0 |
| P2 | NA | 10 | Male | P | DMD | Homo DelEx:50 | Homo DelEx:50 | OUT-OF-FRAME | Xp21.1(31792245-31838252)x0 |
| P3 | NA | 13 | Male | P | DMD | Homo DelEx:45 | Homo DelEx:45 | OUT-OF-FRAME | Xp21.1(31986372-31986636)x0 |
| P4 | NA | 12 | Male | P | DMD | Homo DelEx:46-51 | Homo DelEx:46-51 | OUT-OF-FRAME | Xp21.1(31792031-31950388)x0 |
| P5 | 650 | 34 | Male | P | BMD | Homo DelEx:34-44 | Homo DelEx:34-44 | IN-FRAME | Xp21.1(32234960-32398838)x0 |
| P6 | NA | 10 | Male | P | DMD | Homo DelEx:49-52 | Homo DelEx:49-52 | OUT-OF-FRAME | Xp21.1(31747716-31854946)x0 |
| P7 | NA | 20 | Male | P | DMD | Homo DelEx:51 | Homo DelEx:51 | OUT-OF-FRAME | Xp21.1(31792031-31792352)x0 |
| P8 | 5200 | 12 | Male | P | DMD | Homo DelEx:45-50 | Homo DelEx:45-50 | OUT-OF-FRAME | Xp21.1(31838046-31986636)x0 |
| P9 | NA | 25 | Male | P | BMD | Homo DelEx:45-47 | Homo DelEx:45-47 | IN-FRAME | Xp21.1(31893427-31986636)x0 |
| P10 | 11234 | 9 | Male | P | DMD | Homo DelEx:51-54 | Homo DelEx:51-54 | OUT-OF-FRAME | Xp21.1(31676088-31792352)x0 |
| P11 | 15500 | 12 | Male | P | DMD | Homo DelEx:46-51 | Homo DelEx:46-51 | OUT-OF-FRAME | Xp21.1(31792031-31950388)x0 |
| P12 | NA | 10 | Male | P | DMD | Homo DelEx:48-52 | Homo DelEx:48-52 | OUT-OF-FRAME | Xp21.1(31747716-31893511)x0 |
| P13 | 1400 | 20 | Male | P | BMD | Homo DelEx:45-49 | Homo DelEx:45-49 | IN-FRAME | Xp21.1(31854827-31986636)x0 |
| P14 | NA | 22 | Male | P | DMD | Homo DelEx:46-48 | Homo DelEx:46-48 | OUT-OF-FRAME | Xp21.1(31893258-31950388)x0 |
| P15 | NA | 10 | Male | P | DMD | Homo DelEx:19-45 | Homo DelEx:19-45 | OUT-OF-FRAME | Xp21.1(31986372-32519969)x0 |
| P16 | 15036 | 5 | Male | P | DMD | Homo DelEx:46-51 | Homo DelEx:46-51 | OUT-OF-FRAME | Xp21.1(31792031-31950388)x0 |
| P17 | NA | 14 | Male | P | DMD | Homo DelEx:45 | Homo DelEx:45 | OUT-OF-FRAME | Xp21.1(31986372-31986636)x0 |
| P18 | NA | 18 | Male | P | DMD | Homo DelEx:48-50 | Homo DelEx:48-50 | OUT-OF-FRAME | Xp21.1(31838046-31893511)x0 |
| P19 | 5300 | 13 | Male | P | DMD | Homo DelEx:45-54 | Homo DelEx:45-54 | OUT-OF-FRAME | Xp21.1(31676088-31986636)x0 |
| P20 | 1320 | 14 | Male | P | DMD | Homo DelEx:45-50 | Homo DelEx:45-50 | OUT-OF-FRAME | Xp21.1(31838046-31986636)x0 |
| P21 | NA | 10 | Male | P | DMD | Homo DelEx:45 | Homo DelEx:45 | OUT-OF-FRAME | Xp21.1(31986372-31986636)x0 |
| P22 | 16431.8 | 11 | Male | P | DMD | Homo DelEx:51-55 | Homo DelEx:51-55 | OUT-OF-FRAME | Xp21.1(31645767-31792352)x0 |
| P23 | NA | 15 | Male | P | DMD | Homo DelEx:49-50 | Homo DelEx:49-50 | OUT-OF-FRAME | Xp21.1(31838046-31854946)x0 |
| P24 | NA | 10 | Male | P | DMD | Homo DelEx:45-52 | Homo DelEx:45-52 | OUT-OF-FRAME | Xp21.1(31747716-31986636)x0 |
| P25 | NA | 16 | Male | P | DMD | Homo DelEx:46-47 | Homo DelEx:46-47 | OUT-OF-FRAME | Xp21.1(31947655-31950388)x0 |
| P26 | NA | 15 | Male | P | BMD | Homo DelEx:45-48 | Homo DelEx:45-48 | IN-FRAME | Xp21.1(31893258-31986636)x0 |
| P27 | NA | 20 | Male | P | DMD | Homo DelEx:10-19 | Homo DelEx:10-19 | OUT-OF-FRAME | Xp21.1(32519804-32663284)x0 |
| P28 | 17314.3 | 5 | Male | P | DMD | Homo DelEx:45-52 | Homo DelEx:45-52 | OUT-OF-FRAME | Xp21.1(31747716-31986636)x0 |
| P29 | 27600 | 5 | Male | P | DMD | Homo DelEx:46-51 | Homo DelEx:46-51 | OUT-OF-FRAME | Xp21.1(31792031-31950388)x0 |
| P30 | NA | 13 | Male | P | DMD | Homo DelEx:1-60 | Homo DelEx:1-60 | Difficult to predict | Xp21. 1(31462590-33357743)x0 |
| P31 | 18231.4 | 9 | Male | P | DMD | Homo DelEx:40-45 | Homo DelEx:40-45 | OUT-OF-FRAME | Xp21.1(31986372-32361434)x0 |
| P32 | NA | 13 | Male | P | DMD | Homo DelEx:42-43 | Homo DelEx:42-43 | OUT-OF-FRAME | Xp21.1(32305552-32328421)x0 |
| P33 | 871 | 9 | Male | P | DMD | Homo DelEx:45-52 | Homo DelEx:45-52 | OUT-OF-FRAME | Xp21.1(31747716-31986636)x0 |
| P34 | 18531.2 | 5 | Male | P | DMD | Homo DelEx:49-50 | Homo DelEx:49-50 | OUT-OF-FRAME | Xp21.1(31838046-31854946)x0 |
| P35 | NA | 23 | Male | P | DMD | Homo DelEx:51-55 | Homo DelEx:51-55 | OUT-OF-FRAME | Xp21.1(31645767-31792352)x0 |
| P36 | 7643 | 10 | Male | P | DMD | Homo DelEx:46-51 | Homo DelEx:46-51 | OUT-OF-FRAME | Xp21.1(31792031-31950388)x0 |
| P37 | NA | 9 | Male | P | DMD | Homo DelEx:48-54 | Homo DelEx:48-54 | OUT-OF-FRAME | Xp21.1(31676088-31893511)x0 |
| P38 | 24654 | 7 | Male | P | DMD | Homo DelEx:8-30 | Homo DelEx:8-30 | OUT-OF-FRAME | Xp21.1(32429861-32717415)x0 |
| P39 | 14722 | 14 | Male | P | DMD | Homo DelEx:46-47 | Homo DelEx:46-47 | OUT-OF-FRAME | Xp21.1(31947655-31950388)x0 |
| P40 | 1425 | 14 | Male | P | DMD | Homo DelEx:46-48 | Homo DelEx:46-48 | OUT-OF-FRAME | Xp21.1(31893258-31950388)x0 |
| P41 | 4340 | 10 | Male | P | BMD | Homo DelEx:3-44 | Homo DelEx:3-44 | IN-FRAME | Xp21.1(32173403-32868032)x0 |
| P42 | NA | 25 | Male | P | BMD | Homo DelEx:45-49 | Homo DelEx:45-49 | IN-FRAME | Xp21.1(31854827-31986636)x0 |
| P43 | 21817.5 | 19 | Male | P | DMD | Homo DelEx:48-50 | Homo DelEx:48-50 | OUT-OF-FRAME | Xp21.1(31838046-31893511)x0 |
| P44 | NA | 11 | Male | P | DMD | Homo DelEx:3-19 | Homo DelEx:3-19 | OUT-OF-FRAME | Xp21.1(32509559-33038377)x0 |
| P45 | NA | 15 | Male | P | DMD | Homo DelEx:45 | Homo DelEx:45 | OUT-OF-FRAME | Xp21.1(31986372-31986636)x0 |
| P46 | 250 | 14 | Male | P | BMD | Homo DelEx:3-42 | Homo DelEx:3-42 | IN-FRAME | Xp21.1(32328103-32868032)x0 |
| P47 | 1775 | 14 | Male | P | DMD | Homo DelEx:45-54 | Homo DelEx:45-54 | OUT-OF-FRAME | Xp21.1(31676088-31986636)x0 |
| P48 | NA | 9 | Male | P | DMD | Homo DelEx:45-52 | Homo DelEx:45-52 | OUT-OF-FRAME | Xp21.1(31747716-31986636)x0 |
| P49 | NA | 13 | Male | P | DMD | Homo DelEx:45-52 | Homo DelEx:45-52 | OUT-OF-FRAME | Xp21.1(31747716-31986636)x0 |
| P51 | 2224 | 13 | Male | P | DMD | Homo DelEx:49-50 | Homo DelEx:49-50 | OUT-OF-FRAME | Xp21.1(31838046-31854946)x0 |
| P53 | 3816 | 12 | Male | P | DMD | Homo DelEx:45-54 | Homo DelEx:45-54 | OUT-OF-FRAME | Xp21.1(31676088-31986636)x0 |
| P54 | 213 | 20 | Female | C | DMD | Hetero DelEx:3-7 | Hetero DelEx:3-7 | OUT-OF-FRAME | Xp21.1(32827602-32867919)x1 |
| P55 | 335 | 8 | Male | P | BMD | Homo DelEx:3-41 | Homo DelEx:3-41 | IN-FRAME | Xp21.1(32360182-32868032)x0 |
| P57 | 228 | 15 | Female | C | DMD | Hetero DelEx:45-52 | Hetero DelEx:45-52 | OUT-OF-FRAME | Xp21.1(31747716-32173507)x1 |
| P58 | 679 | 10 | Male | P | DMD | Homo DelEx:45-52 | Homo DelEx:45-52 | OUT-OF-FRAME | Xp21.1(31747716-31986636)x0 |
| P61 | 2951 | 11 | Male | P | DMD | Homo DelEx:46-55 | Homo DelEx:46-55 | OUT-OF-FRAME | Xp21.1(31645767-31950388)x0 |
| P62 | 1656 | 12 | Female | C | DMD | Hetero DelEx:2-10 | Hetero DelEx:2-10 | OUT-OF-FRAME | Xp21.1(32591846-33038377)x1 |
| P66 | 2713 | 12 | Male | P | DMD | Homo DelEx:51 | Homo DelEx:51 | OUT-OF-FRAME | Xp21.1(31792031-31792352)x0 |
| P71 | NA | 16 | Female | C | DMD | Hetero DelEx:46-49 | Hetero DelEx:46-49 | OUT-OF-FRAME | Xp21.1(31854827-31950388)x1 |
| P72 | NA | 35 | Female | C | DMD | Hetero DelEx:46-48 | Hetero DelEx:46-48 | OUT-OF-FRAME | Xp21.1(31893343-31986460)x1 |
| P74 | 3348 | 15 | Male | P | DMD | Homo DelEx:46-49 | Homo DelEx:46-49 | OUT-OF-FRAME | Xp21.1(31854827-31950388)x0 |
| P78 | 2589 | 14 | Male | P | BMD | Homo DelEx:49-50 | Homo DelEx:49-50 | IN-FRAME | Xp21.1(31838046-31854946)x0 |
| P80 | 3252 | 12 | Male | P | DMD | Homo DelEx:46-50 | Homo DelEx:46-50 | OUT-OF-FRAME | Xp21.1(31838046-31950388)x0 |
| P82 | 2977 | 13 | Male | P | DMD | Homo DelEx:49-52 | Homo DelEx:49-52 | OUT-OF-FRAME | Xp21.1(31747716-31854946)x0 |
| P84 | 1784 | 34 | Male | P | BMD | Homo DelEx:3-4 | Homo DelEx:3-4 | IN-FRAME | Xp21.1(32862799-32868032)x0 |
| P87 | 4124 | 9 | Male | P | DMD | Homo DelEx:46-47 | Homo DelEx:46-47 | OUT-OF-FRAME | Xp21.1(31947655-31950388)x0 |
| P88 | 18840 | 5 | Male | P | DMD | Homo DelEx:46-50 | Homo DelEx:46-50 | OUT-OF-FRAME | Xp21.1(31838046-31950388)x0 |
| P93 | 2020 | 13 | Male | P | DMD | Homo DelEx:46-55 | Homo DelEx:46-55 | OUT-OF-FRAME | Xp21.1(31645767-31950388)x0 |
| P101 | 3544 | 11 | Male | P | DMD | Homo DelEx:8-10 | Homo DelEx:8-10 | OUT-OF-FRAME | Xp21.1(32662161-32717415)x0 |
| P104 | 1339 | 9 | Male | P | DMD | Homo DelEx:45-52 | Homo DelEx:45-52 | OUT-OF-FRAME | Xp21.1(31747716-31986636)x0 |
| P106 | 659 | 34 | Male | P | BMD | Homo DelEx:3-4 | Homo DelEx:3-4 | IN-FRAME | Xp21.1(32862799-32868032)x0 |
| P107 | 2080 | 8 | Male | P | DMD | Homo DelEx:51 | Homo DelEx:51 | OUT-OF-FRAME | Xp21.1(31792031-31792352)x0 |
| P108 | 259 | 24 | Male | P | DMD | Homo DelEx:10-19 | Homo DelEx:10-19 | OUT-OF-FRAME | Xp21.1(32519804-32663284)x0 |
| P111 | 70 | 32 | Female | C | BMD | Hetero DelEx:47-49 | Hetero DelEx:47-49 | IN-FRAME | Xp21.1(31838046-31947867)x1 |
| P112 | 277 | 8 | M | P | DMD | Homo DelEx:46-51 | Homo DelEx:46-51 | OUT-OF-FRAME | Xp21.1(31792031-31950388)x0 |
| P123 | 104 | 27 | Female | C | BMD | Hetero DelEx:49-50 | Hetero DelEx:49-50 | IN-FRAME | Xp21.1(31838046-31854946)x1 |

P, Patient; C, Carrier; DMD, Duchenne Muscular Dystrophy; BMD, Becker Muscular Dystrophy; CPK, Creatine Phosphokinase; Ex, Exon; Del, deletion, Hetero; Heterozygous, Homo; Homozygous, NA, not available.
